# Rose Gana Fomban Leke: rethinking malaria

**DOI:** 10.2471/BLT.22.030122

**Published:** 2022-01-01

## Abstract

Rose Gana Fomban Leke talks to Gary Humphreys about rethinking approaches to malaria elimination and her efforts to support the next generation of women scientists in Cameroon.

*Q: How did you come to your focus on parasitology?*

A: I grew up in rural Cameroon and had some fairly formative encounters with parasites early on. I had regular bouts of malaria and remember my mother giving me a kind of steam treatment with herbs that she would put into a boiling pot of water and then put a blanket over me to get me to sweat which actually makes sense in terms of boosting cytokine activity, although we had no idea about any of that back then. I also had a lung abscess when I was seven and that required lengthy surgery. I think those experiences probably helped put me on the path of science and eventually studying for my master’s at the University of Illinois at Urbana-Champaign in the 1970s under Dr Paul Silverman, who was one of the pioneers in the development of a malaria vaccine. I also have to credit my mother and father. My father was a headmaster and really pushed me to work hard and encouraged me to be a scientist or a doctor. My mother, who had not been to school herself, did everything at home to make sure I had the time to study.

*Q: So, you left Cameroon to study in the USA?*

A: In 1966. I sat for the examination for a scholarship through the African Scholarship Program of American Universities (ASPAU) and of the 18 who passed, was the only woman. After the USA, I did a doctorate in parasitology at the University of Montreal where I was also very much in the minority. And here we are, more than 50 years later, women are still in the minority in scientific research, particularly at the higher levels of academia.

*Q: You are trying to change that with the Higher Institute for Growth in Health Research for Women (HIGHER Women) in Cameroon. Can you tell us a little about that initiative?*

A: I set it up with some colleagues in 2015. Its core activity is a mentor–protégé programme that puts together established female scientists who share their experience and expertise with female protégés who typically have less than five years of research experience. This sharing is not limited to advice about how to conduct research, write papers or apply for grants and so forth, but also focuses on soft skills such as time management, how to balance life and career and so on. It can be very difficult for women to do this especially once they get married and have children. A lot of women get discouraged, but I am proof that it can be done! I have four children and yet have managed to pursue a career which has included working full-on with polio and malaria elimination programmes, teaching, and publishing over a 100 peer-reviewed articles. So, we try to show them how to deal with all that, and also encourage connection through workshops and social events and via different web platforms. To date we have partnered 18 mentors with 103 protégés from 15 research institutions and universities across Cameroon. I personally have four protégés under my wing. Many of our protégés have gone on to successful careers, publishing peer-reviewed articles, talking at conferences, winning awards. But society changes slowly, as do social norms. Gender bias is still a significant impediment to women advancing in Cameroon as in many other countries. Even here in the University of Yaoundé you find women coming into the programmes, but as you go up the hierarchy you encounter them less and less. Among the academicians in Cameroon today, women make up 12.5% of associate professors and only 7% of full professors.

“[Malaria data] has to be valued for what it is – actionable intelligence.”

*Q: You mention your work on malaria elimination, another field in which progress has been slow. According to last month’s World Malaria Report there were an estimated 241 million malaria cases in 2020, up from 227 million in 2019. Prior to that, estimated case incidence had barely changed since 2015. What needs to happen in 2022 to regain momentum?*

A: That’s a question that a lot of experts have been thinking about for some time and was a focus of the discussions organized by Harvard University as part of the Rethinking Malaria initiative.

*Q: Can you tell us more about that initiative?*

A: It was a year-long global consultation that brought together over 200 stakeholders to share their views and discuss new approaches to tackling malaria. The discussion included thought leaders but was rooted in listening and learning from people on the frontlines of malaria control – especially those individuals in high-burden countries, most of which are in sub-Saharan Africa. The key findings were shared at a virtual forum that took place in September and which I chaired. The initiative is ongoing, and I am hoping to see further consultation in 2022.

*Q: What were the main take-aways of the September forum?*

A: As you’d expect for such a complex issue there were a lot, ranging from the fact that malaria is a societal problem, a problem of development, and not just a medical problem, that malaria surveillance remains woefully inadequate and the quality of the data generated is questionable and generally ignored or at least underused. It also became clear that despite the disease burden imposed on their populations, national governments still fail to mobilize and effectively channel the necessary resources into malaria as part of essential health service provision. For me, Health for All means tackling the problem of malaria as a part of universal health coverage (UHC), which means integrating prevention, diagnosis and treatment into basic health-care packages, as well as encouraging and supporting community participation, notably in regard to monitoring and surveillance.

*Q: Can you give an example of rethinking that came out of the discussions?*

A: New thinking around how we collect, share and use data is one example. The lack of quality data has been a major impediment to advancing malaria elimination. Everyone at the forum agreed on this, and there was some interesting discussion of how we can do a better job in this area. Properly resourced primary health care can play a key role in identifying cases and sharing information, which goes back to what I said about malaria being integrated into UHC programmes. Community involvement, participation and ownership were also discussed as very important components. More can be done to bring communities into the data gathering space, especially where people have access to cell phones. This has already been demonstrated in efforts to eradicate polio which resulted in Africa being declared wild poliovirus-free in August of 2020. I am thinking of initiatives using the SMS-based application AVADAR, which was used for reporting, monitoring and surveillance of polio cases. So, data gathering is crucial, but what is done with that data is just as important. It cannot just be generated and shelved. It has to be valued for what it is – actionable intelligence. One possible application of data being discussed is the generation of malaria stratification maps showing the way cases, deaths, access to health services and malaria interventions intersect. Such maps can then be used to target interventions.

Of course, the benefits of engaging local communities in malaria monitoring are not limited to data generation. For me, one of the key take-aways of the discussion was the need to get communities thinking about malaria and understanding that they don’t have to accept malaria as just a fact of life. There are now compelling examples of countries that have rolled back malaria to the point of elimination, the most obvious being China which was reporting 30 million cases of malaria annually in the 1940s and has been declared malaria-free today. People will say China is China and that in Africa we have a lot of people living in poverty in remote rural areas, but I still believe we can achieve a great deal on the continent by engaging with the communities.

“The pandemic has […] shone a light on new vaccine research and development technologies.”

*Q: In what way is the COVID-19 pandemic informing the rethinking of malaria discussion?*

A: The pandemic has seen the wholesale reallocation of resources that have impacted many national and international malaria initiatives, but there are some important silver linings. For example, it has shone a light on the various shortcomings of national health-care systems and has led to some capacity-building in certain areas – notably the diagnostic capacity of health centres. This needs to be carried forward and will hopefully lead to a reprioritization of health-care funding by governments. The pandemic has also shone a light on the new vaccine research and development technologies, including nucleotide-based technologies and it is to be hoped that they can be applied in future malaria vaccine research. Not that malaria vaccine research and development wasn’t going on before the pandemic. The development of the RTS,S vaccine (the first malaria vaccine to be endorsed by WHO (World Health Organization)) is proof of that. But the highlighting of mRNA and other platform technologies has got everyone’s attention. We will see what comes down the pipeline from Professor Adrian Hill and Doctor Stephen Hoffman among others in the coming year or two – but for now we need to get the RTS,S vaccine into the EPI (Expanded Program on Immunization) programme so that it can start saving lives and help us regain momentum on rolling back malaria.

*Q: Do you think that a year from now we will be lamenting another year without progress on malaria prevalence?*

A: Let me be optimistic. My hope is that the recommendations that came out of the Rethinking Malaria initiative, the global interactions that have followed and the compelling involvement of WHO will lead to a revival and strengthening of efforts in the fight against malaria. However, it is crucial that the high-burden countries also step up their efforts, funding malaria prevention and treatment and engaging with the communities to make sure that they buy into the idea that something can be done. Because something can be done and there is no reason to wait to do it. Action is needed now!

